# Chemical Composition, Protective Effects, and Mechanisms of Volatile Oil from Fructus Gleditsiae Abnormalis with Nasal Administration against Ischemic Injury in HFD and MCAO-Induced Rats

**DOI:** 10.1155/2021/8880996

**Published:** 2021-11-01

**Authors:** Chengyue Luo, Yumei Wu, Xiaolan Chen, Wei Han, Jing Wan, Nana Dong, Bili Deng, Dereje Kebebe

**Affiliations:** ^1^College of Pharmaceutical Sciences, Guizhou University of Traditional Chinese Medicine, Guiyang 550025, China; ^2^Department of Pharmaceutics, School of Pharmacy, Zunyi Medical University, Zunyi 563000, China; ^3^School of Pharmacy, Institute of Health Sciences, Jimma University, Jimma, Ethiopia

## Abstract

Fructus Gleditsiae Abnormalis (FGA) has been used as a traditional Chinese medicine (TCM) for the treatment of stroke caused by phlegm and blood stasis. However, its substance basis and mechanism of action are currently unknown. This study is aimed to analyze the constituents of the volatile oil in FGA (VOFGA) using gas chromatography coupled with mass spectrometry (GC-MS) and explore the underlying effects and mechanisms of VOFGA in the prevention and treatment of ischemia stroke. An *in vivo* ischemia model was constructed by combination treatment of high-fat diet (HFD) and middle cerebral artery occlusion (MCAO) method. After administration, the cerebral infarction volume, the brain water content, hemorheology, blood lipids, inflammatory factors, oxidative stress indicators, Bax, Bcl-2, and cleaved caspase-3 and histological examination (HE) were determined and observed to explore the underlying effects and mechanisms of VOFGA against ischemia stroke. The results showed that forty components were determined after analyzed by GC-MS, and the percentage content of palmitate, paeonol, violetone, linalool, salpinol, citral, and methyleugenol were 4.69%, 5.2%, 3.56%, 3.31%, 2.42%, 2.65%, and 1.67%, respectively. The high dose of VOFGA could inhibit neurological damage; reduce the cerebral infarction volume and brain water content; improve whole blood viscosity and red blood cell aggregation index at various shear rates; reduce the levels of TG, TC, LDL-C, TNF-*α*, IL-1*β*, MDA, and NO; increase the contents of HDL-C, IL-10, and SOD; downregulate the expressions of Bax and cleaved caspase-3 in the ischemic regions; and upregulate the expressions of Bcl-2. These effects implied that VOFGA may exert neuroprotective effects via inhibiting ischemia-triggered oxidative damage-regulating blood lipid factors and reducing the production of proinflammatory mediators against cerebral I/R injury and neuronal apoptosis. The VOFGA presents a potential treatment value for cerebral ischemic stroke, and it may offer insights into discovering new active compounds for the treatment of ischemic stroke.

## 1. Introduction

Stroke is one of the leading causes of global disability and death [1], which the aging of the global population will lead to an increase [2]. The World Health Organization estimates that without more population interventions, the incidence of primary stroke is expected to increase to 23 million per year by 2030 [3]. Cerebral ischemia is the main type of stroke, which accounting for 60% to 80% of stroke cases [4]. The theory of TCM holds that stroke is, on the basis of internal injury, combined with the imbalance between internal organs and Yin and Yang, caused by irregular diet, unsmooth emotions, excessive fatigue, etc. According to TCM, the pathogenesis of stroke is deficiency, mainly due to the mutual knot of phlegm and blood stasis blocking the meridians, causing the Qingqiao to be blocked. Phlegm and blood stasis are theoretically believed to be intermingled with blood stasis and phlegm dampness, and the two are the direct pathogenic factors, which are the key pathogenesis of stroke. At the same time, phlegm and blood stasis are pathological products with similar causes [5]. Therefore, the current TCM treatment methods for stroke are mainly expectorant and resuscitation. However, due to the narrow time window for stroke treatment, it is of great significance for the prognosis of patients if they receive first aid during the acute attack.

FGA is a dry, sterile fruit of *Gleditsia sinensis* Lam., which has been included in the Chinese Pharmacopoeia (CP) since 1995. It mainly contains a variety of chemical components such as saponins, flavones, and volatile oils and has anti-inflammatory, antitumor, and antiallergic effects. The CP recorded that the powder of FGA was used via nasal administration to treat the teeth coma and unsmooth sputum, which is considered to be caused by stroke [6]. Studies showed that the nasal duct is the fastest and the most direct route to the brain and the drug passes from the blood-brain barrier in a noninvasive manner from this way [7]. So far, many works have showed that intranasal administration can significantly improve brain targeting efficiency and bioavailability and significantly decrease infarct size and improvement of behavioral outcomes were observed in MCAO rat models [8]. However, no pharmacological experimental studies have been conducted on the treatment of stroke by nasal route administration of FGA. From the CP, it can be speculated that the effective part of FGA in the treatment of stroke may be volatile components, which laid a theoretical foundation for the analysis and pharmacological effects of volatile oil components and also provided a basis for the nasal administration in our study.

To that end, this work aimed to analyze the constituents of VOFGA by GC-MS. An *in vivo* model of stroke caused by intermingled phlegm and blood stasis was constructed by high-fat feeding combined with middle cerebral artery occlusion method. After administration, the brain water content, hemorheology, blood lipids, inflammatory factors, oxidative stress indicators, Bax, Bcl-2, and cleaved caspase-3 and histological examination (HE) were determined and observed. The ultimate goal is to evaluate the effect and mechanism of VOFGA in the treatment of stroke caused by intermingled phlegm and blood stasis and provide a certain experimental basis for the development and application of nasal administration preparations of VOFGA.

## 2. Materials and Methods

### 2.1. Chemicals and Resources

Protein quantification kit, total superoxide dismutase assay kit, malondialdehyde kit, and nitric oxide assay kit were purchased from Nanjing Jiancheng Bioengineering Institute. Rat IL-1*β* ELISA kit, Rat IL-10 ELISA kit, and Rat TNF-*α* ELISA kit were supplied by Shanghai Zhuocai Biotechnology Co. FGA was obtained from Beijing Tongrentang Medicine Corporation (Beijing, China; batch No. 20170408).

### 2.2. Animals

Male Sprague-Dawley (SD) rats, weighing from 250 g to 280 g, were purchased from Chongqing Tengxin Biotechnology Co. The animals were housed under controlled conditions (25 ± 2°C and 12-h light-dark cycle), allowed free movement, and were given food and water ad libitum. The experiments were performed in accordance with the guideline of the Animal Care Committee of the Guizhou University of Traditional Chinese Medicine.

### 2.3. Extraction of Volatile Oil

100 grams of FGA was weighed, then 10 mL of n-hexane and 1000 mL of water were added to it, and then the mixture was subjected to steam distillation for 10 h. Finally, the n-hexane fraction was collected and dried, and then the VOFGA was obtained.

### 2.4. Analysis of Main Components of VOFGA

Analysis of the compounds in the volatile oil was performed on Agilent 6890N gas chromatograph (GC) combined with a 5975B mass spectrometer (MS) equipped with a HP-5MS column (30 m × 0.25 mm × 0.25 *μ*m). High-purity helium was used as the carrier gas at a constant flow rate of 1.0 mL/min, and the inlet temperature was 250°C. The temperature-increasing procedure was set as follows: started at 40°C and maintained for 3 min, and then raised to 160°C at a rate of 3°C/min and kept it for 2 min; it was then raised to 220°C at a rate of 8°C/min and held for 3 min. The detection of the volatile oil was in the full-scan mode with the ion source temperature of 230°C and quadrupole temperature of 180°C. The collected mass spectrogram was searched using the NIST spectral database to identify the components in the volatile oil, and the relative contents of each component were analyzed by the area normalization method.

### 2.5. Establishment of the HFD and MCAO-Induced Model

The establishment of the model of focal cerebral ischemia/reperfusion with intermingled phlegm and blood stasis syndrome (IPBSS) was done by high-fat feeding combined with MCAO.

After 70 SD male rats were fed with ordinary diet for 7 days, they were randomly housed in different cages, each cage containing 5 rats; of which 1 cage was continued to be fed with a normal diet and the remaining cages were given HFD for 28 days [9, 10].

The MCAO model was prepared by the suturing method [11]. The rats were anesthetized by continuous isoflurane gas and placed on a heating pad set at 37°C to maintain the rats' body temperature throughout the experiment. After a midline neck incision, tissues and muscles were separated from the right common carotid artery, and then the external carotid artery (ECA) and internal carotid artery (ICA) were found. A small incision was made in the common carotid artery 4 mm away from the bifurcation, and a suture (diameter 0.25 mm, head diameter 0.30 ± 0.02 mm) was introduced into the ECA lumen and extended into the ICA (18.5 ± 0.5 mm) to block the origin of the middle cerebral artery (MCA). After 2 h of ischemia, the suture was gently pulled out from the brain to the bifurcation port and then reperfused for 24 h. Blood and brain tissues were taken and various indicators were measured.

Blood was taken from the fundus venous plexus, and the blood lipids of the rats were detected. If abnormal indicators were detected, the modeling of hyperlipidemia was considered successful.

### 2.6. Groups and Drug Administration

The rats successfully established in the hyperlipidemia model were randomly divided into a sham operation group, a model control group, a nimodipine group (5 mg/kg), no-dose VOFGA group (0.0 mg/kg), low-dose VOFGA group (0.077 mg/kg), medium-dose VOFGA group (0.154 mg/kg), and high-dose VOFGA group (0.308 mg/kg). The normal, sham operation, and model groups were given normal saline through the nasal cavity once a day for 7 consecutive days, and the model of focal cerebral ischemia/reperfusion was made 1 h after the last dose.

### 2.7. Measurement of Neurological Deficit

After 24 h of reperfusion in rats, the Berderson scoring method was adopted [12]. A score of 0 is normal, and there is no neurological defect; a score of 1 is the left forelimb flexed when lifting the tail; a score of 2 is a turn to the left; a score of 3 is a dump to the left; a score of 4 is unable to walk and is unconscious.

### 2.8. Detection of Brain-Water Content and Infarct Volume

The brain tissue was accurately weighed for wet weight and then dried in a 105°C electric blast drying oven to constant weight, weighed, and calculated the water content of the brain tissue.

The infarct volume was assessed 24 h after MCAO. Brain tissue was removed and frozen at −20°C for 20 min and then cut into 2 mm-thick coronal sections (6 slices). The sections were placed in 2% triphenyl-2,3,5-tetrazoliumchloride (TTC) at 37°C for 20 min. Each section was soaked in 4% paraformaldehyde for 24 h, and then images were collected for further analysis. ImageJ software was used to analyze the infarct area.

### 2.9. Measurement of Hemorheology Indicators

After blood was taken from the abdominal aorta, blood rheology was measured using a blood viscosity meter within 4 h.

### 2.10. Determination of Blood Lipids and Inflammatory Factors

Another 2 mL of blood was taken and left to stand at 4°C for half an hour, centrifuged at 3000 r/min for 10 min, and the serum was separated. Cholesterol (TC), triglyceride (TG), low-density lipoprotein cholesterol (LDL-C), HDL-C, IL-1*β*, IL-10, and TNF-*α* were measured according to the procedure of the kit.

### 2.11. Detection of Oxidative Stress Indicators

The ischemic side of the brain was weighed and made into a 10% homogenate under an ice bath, which was centrifuged at 4°C 3000 r/min for 10 min. The supernatant was taken to measure the contents of SOD, MDA, and NO according to the instructions of the kit.

### 2.12. Western Blotting Analysis

After the rats were reperfused for 24 hours, the brain tissue were homogenized and lysed with RIPA buffer and then centrifuged at 12000 r/min for 5 min at 4°C, and the supernatant was taken. The total protein concentration was measured by the BCA kit, and the samples of each group were electrophoresed and transferred to the membrane by the wet transfer method. After blocking with 5% nonfat milk, the PVDF membranes were incubated with the primary antibodies cleaved caspase-3 (1 : 2000, Catalog No. AF7022; Affinity Biosciences Ltd., USA) and *β*-actin (1 : 500, Catalog No. BM0627; Boster Biological Technology Co. Ltd., USA) overnight at 4°C. After being washed with the Tris-buffered saline-Tween 20 (TBST) buffer five times, the PVDF membranes were incubated with peroxidase-conjugated secondary antibodies. The membranes were then washed with TBST five times, and the proteins were visualized with an ECL detection solution (Catalog No. P1050; Applygen Technologies Inc., Beijing, China). The intensity of the protein bands was quantified with ImageJ software, and each test was repeated at least three times.

### 2.13. Histological Examination

In order to better observe the microscopic changes of the ischemic lateral brain tissue, the tissues were soaked in 4% paraformaldehyde and subjected to HE staining. Later, all the slides were observed under a microscope.

### 2.14. Statistical Analysis

SPSS 17.0 software was used for statistical analysis. All measurement data were expressed as means ± standard deviation, and one-way analysis of variance (ANOVA) in repeated measurement was adopted for comparison among multiple groups. *P* < 0.05 was considered statistically significant.

## 3. Results

### 3.1. Analysis of Main Components in VOFGA

**Figure 1:**
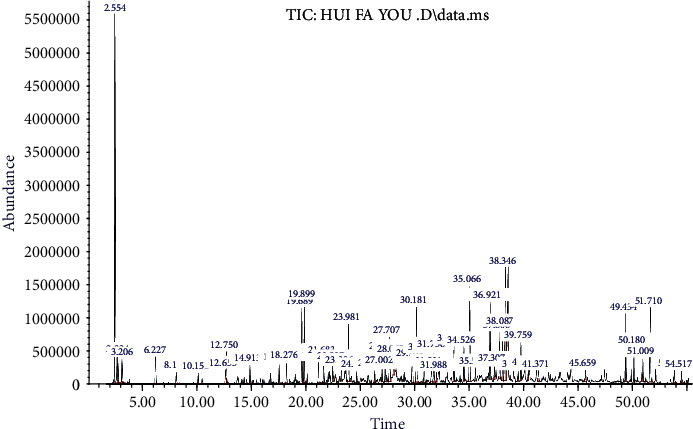
The total ion chromatogram of VOFGA.

As shown in Figure 1, the total ion chromatogram of the composition of VOFGA was obtained by using a GC-MS spectrometer. Forty components were determined after the analysis, and the results are shown in Table 1. According to the analysis, the percentage content of palmitate, paeonol, violetone, linalool, salpinol, citral, and methyleugenol were 4.69%, 5.2%, 3.56%, 3.31%, 2.42%, 2.65%, and 1.67%, respectively. Among these, methyleugenol and linalool have been proven to have a protective effect for the central nervous system after focal ischemia [13, 14].

### 3.2. Effects of VOFGA on Neuroethology

It can be seen from Table 2, as compared with the normal group, the neurobehavioral score of the model group was significantly different (*P* < 0.01), indicating that the model was successfully developed. Compared with the model group, there was a significant difference in each administration group (*P* < 0.01). The group that received high dose of volatile oil exerted an effect superior to the other administration groups and slightly more than the nimodipine group.

### 3.3. Effects of VOFGA on Cerebral Edema and Infarct Volume

**Figure 2:**
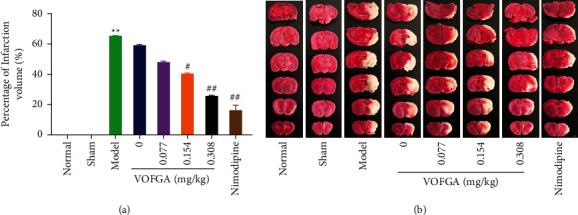
Effects of different doses of VOFGA treatment on infarction volume (a) and representative sections of TTC staining in normal rats, rats treated with the sham operation, ischemia model rats, and the same model rats treated with 0, 0.077, 0.154, 0.308 mg/kg of VOFGA and nimodipine, respectively (b). Compared with the normal group,  ^*∗*^*P* < 0.05,  ^*∗∗*^*P* < 0.01. Compared with the model group, ^#^*P* < 0.05, ^##^*P* < 0.01.

As can be seen from Table 2 and Figure 2, the brain water content and the percentage of infarction volume in the model group were significantly higher than those of the normal group (*P* < 0.01), indicating that the model was successfully developed. Compared with the model group, the water content in the brain tissue of rats in all administration groups was significantly reduced (*P* < 0.01). The group that received high dose of volatile oil exerted an effect superior to the other administration groups and comparable to the nimodipine group.

As shown in Figure 2, after 24 hours of reperfusion, in addition to the normal group and the sham operation group, the rats in the other groups had different degrees of infarction. Among them, compared with the normal group, a significantly higher percentage of infarction volume of rats in the model group was observed (*P* < 0.01). Compared with the model group, a significantly lower percentage of infraction was exhibited by the high-dose volatile oil group and the nimodipine group (*P* < 0.01). Therefore, the finding shows that the high-dose volatile oil and nimodipine have a certain effect on cerebral ischemia-reperfusion injury in rats.

### 3.4. Determination of Hemorheology

As shown in Table 3, compared with the normal group, the sham operation group and the model group had significantly increased whole blood viscosity (WBV) and Carson viscosity (KS) at various shear rates (*P* < 0.01), and the erythrocyte aggregation index (HJJ) significantly increased (*P* < 0.01), indicating that the modeling was successful. In contrast to the model group, the whole blood viscosity and the red blood cell aggregation index of the nimodipine group and all administration groups at each shear rate were significantly reduced (*P* < 0.01). The group that received high dose of volatile oil exerted an effect superior to the other administration groups and comparable to the nimodipine group.

### 3.5. Effect of VOFGA on Serum Lipids in Rats

**Figure 3:**
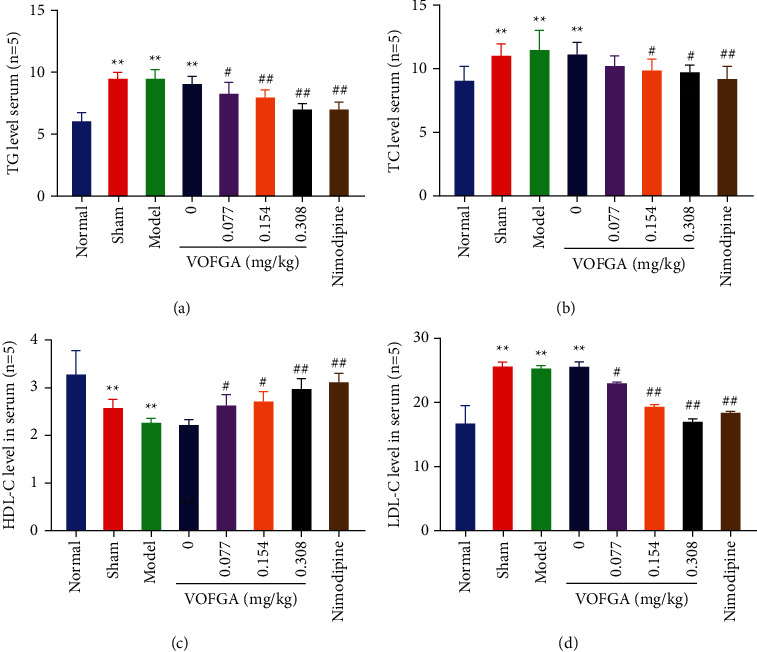
The TG, TC, HDL-C, and LDL-C levels in normal rats, rats treated with the sham operation, ischemia model rats, and the same model rats treated with 0, 0.077, 0.154, 0.308 mg/kg of VOFGA and nimodipine, respectively. Compared with the normal group,  ^*∗*^*P* < 0.05,  ^*∗∗*^*P* < 0.01. Compared with the model group, ^#^*P* < 0.01, ^##^*P* < 0.01.

As represented in Figure 3, the levels of TG, TC, and LDL-C in the sham-operated and model groups were significantly higher than those in the normal group (*P* < 0.01), while the levels of HDL-C were significantly lower than those in the normal group (*P* < 0.01), indicating that the modeling was successful. Compared with the model group, except for the TC level in the low-dose volatile oil group, the levels of TG, TC, and LDL-C in the nimodipine group and the medium-dose and high-dose volatile oil groups were significantly reduced (*P* < 0.05), whereas the HDL-C level was significantly higher than that of the model group (*P* < 0.05). In short, the volatile oil exerts the effect in a dose-dependent manner. The group that received the high dose of volatile oil showed a better effect than the medium-dose and low-dose drug groups and a slightly better effect than the nimodipine group.

### 3.6. Effect of VOFGA on TNF-*α*, IL-10, and IL-1*β* in Rat Serum

**Figure 4:**
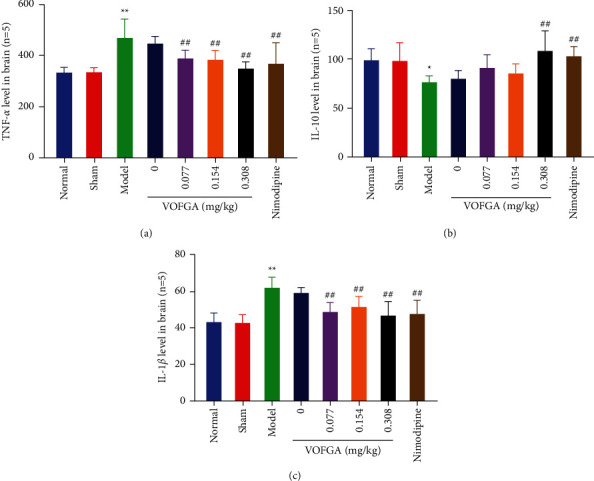
The TNF-*α*, IL-10, and IL-1*β* levels in normal rats, rats treated with the sham operation, ischemia model rats, and the same model rats treated with 0, 0.077, 0.154, 0.308 mg/kg of VOFGA and nimodipine, respectively. Compared with the normal group,  ^*∗*^*P* < 0.05, ^*∗∗*^*P* < 0.01. Compared with the model group, ^#^*P* < 0.05, ^##^*P* < 0.01.

As shown in Figure 4, compared with the normal group, a significantly (*P* < 0.01) higher serum level of TNF-*α* and IL-1*β* and considerably lower levels of IL-10 were observed in the model group (*P* < 0.01). However, in comparison with the model group, the contents of TNF-*α* and IL-1*β* in the serum of the nimodipine group and the low-dose, medium-dose, and high-dose volatile oil groups were significantly low (*P* < 0.01), while the contents of IL-10 in the serum of the nimodipine group and the high-dose volatile oil group were significantly low (*P* < 0.01); the efficacy of the high-dose volatile oil group was slightly higher than that of the nimodipine group.

### 3.7. Effect of VOFGA on SOD, MDA, and NO

**Figure 5:**
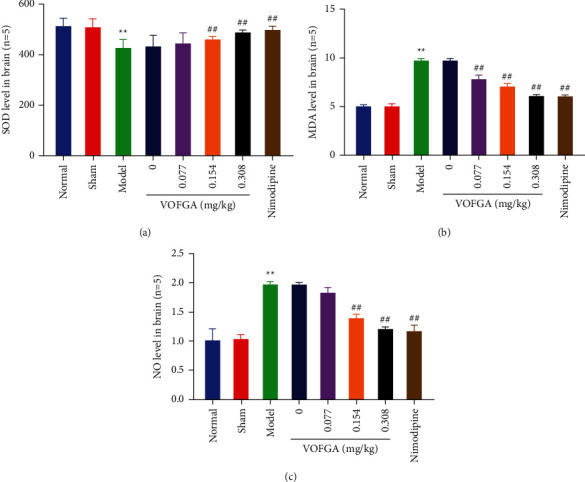
SOD, MDA, and NO in normal rats, rats treated with the sham operation, ischemia model rats, and the same model rats treated with 0, 0.077, 0.154, 0.308 mg/kg of VOFGA and nimodipine, respectively. Compared with the normal group,  ^*∗*^*P* < 0.05, ^*∗∗*^*P* < 0.01. Compared with the model group, ^#^*P* < 0.05, ^##^*P* < 0.01.

As shown in Figure 5, compared with the model group, in the medium-dose and high-dose volatile oil groups, the level of SOD significantly increased, whereas the levels of MDA and NO in rat brain tissues reduced (*P* < 0.01).

### 3.8. Effect of VOFGA on Bax, Bcl-2, and Cleaved Caspase-3

**Figure 6:**
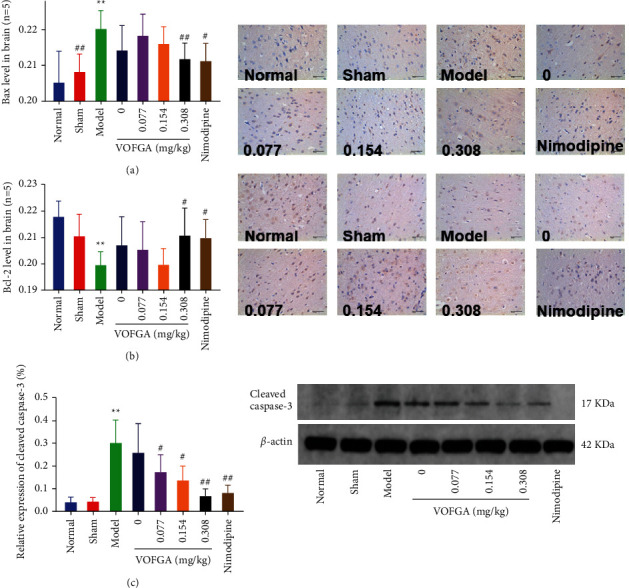
Bax, Bcl-2, and cleaved caspase-3 in normal rats, rats treated with the sham operation, ischemia model rats, and the same model rats treated with 0, 0.077, 0.154, 0.308 mg/kg of VOFGA and nimodipine, respectively. The magnification was ×400. Compared with the normal group,  ^*∗*^*P* < 0.05,  ^*∗∗*^*P* < 0.01. Compared with the model group, ^#^*P* < 0.05, ^##^*P* < 0.01.

Compared with the normal group, the levels of Bax and cleaved caspase-3 protein in the brain tissue of the model group were significantly increased, while the Bcl-2 protein content was significantly reduced (*P* < 0.01). Compared with the model group, the levels of cleaved caspase-3 and Bax protein in the brain tissues of the nimodipine group and high-dose volatile oil group decreased significantly (*P* < 0.05), and the levels of Bcl-2 protein increased (*P* < 0.05). This suggests that high-dose volatile oil can produce the effect equivalent to nimodipine (Figure 6).

### 3.9. Histological Examination

**Figure 7:**
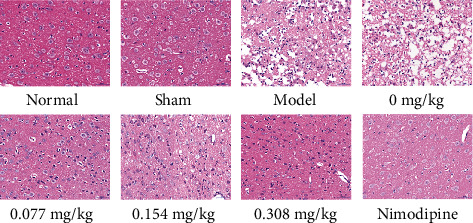
Histological examination of brain tissue. The images of H/E staining (×400). The histological examination of brain tissue from normal rats, rats treated with the sham operation, ischemia model rats, and the same model rats treated with 0, 0.077, 0.154, 0.308 mg/kg of VOFGA and nimodipine, respectively. The magnification was ×400.

The result of HE showed that in the normal group, the sham operation group, and the nimodipine-positive drug group, the morphological structure of brain tissue was normal. However, in the model group and the solvent group, the morphology of nerve cells is not clear, the nucleus is dissolved or disappeared, the cell membrane is fragmented, and the nerve fibers are arranged disorderly and irregularly. In the low-dose, medium-dose, and high-dose volatile oil groups, the brain tissue structure was normal, and the nerve cells were in a regular arrangement, which showed clear outlines with rounded nuclei and clear, visible nucleoli. The intact nerve cells were in a tight arrangement and uniformly colored. All the above results got closer to the normal group, the sham operation group, and the nimodipine-positive drug group. Overall, the low-dose, medium-dose, and high-dose volatile oil groups can improve the pathological changes to some extent (Figure 7).

## 4. Discussion

Hemorheology mainly studies the fluidity and deformation of blood and its constituents. It particularly focused on the research areas including rheology, viscosity, aggregation, and coagulation of blood in blood vessels [15, 16]. Understanding of blood fluidity and its changes under physiological or pathological conditions has a significant impact on certain diseases, especially on cardiovascular and cerebrovascular diseases related to stroke [17]. Clinical hemorheology test indicators include whole blood viscosity, plasma viscosity, hematocrit, and red blood cell aggregation index. An increase in the red blood cell aggregation index means that the aggregation is enhanced, the blood fluidity is weakened, it is easy to cause blood perfusion disorders, and it is easy to form thrombus, which leads to tissue or organ ischemia and hypoxia. The results of clinical studies show that the hemorheology index of patients with ischemic stroke is significantly higher than that of healthy people [18]. This study showed that the whole blood viscosity and the red blood cell aggregation index of the nimodipine group and all administration groups at each shear rate were significantly reduced (*P* < 0.01).

Many epidemiological studies have confirmed that dyslipidemia is a risk factor for cardiovascular disease morbidity and mortality in the general population. Blood lipid is an essential substance for the body's cell metabolism. It is involved in the body's energy metabolism and the synthesis of cytoplasmic membranes, steroid hormones, and bile acids. TG is a component of lipids, whose function is to supply and store energy, and LDL-C is a lipoprotein particle that carries cholesterol into peripheral tissue cells and can be oxidized to oxidize LDL-C [19]. However, excessive levels of oxidized LDL-C can accumulate in the arterial wall, and an increase in both levels can lead to worsening of acute ischemic stroke. There is a positive correlation between serum TC levels and cardiovascular disease risk in the general population. Plasma LDL concentration is a well-studied risk factor for ischemic stroke. This study suggested that the volatile oil exerts the effect in a dose-dependent manner, which high-dose volatile oil could reduce the levels of TG, TC, and LDL-C and increase the level of HDL-C.

Inflammation is an important cause of secondary injury after cerebral ischemia reperfusion, and it is also an important pathogenesis [20]. When cerebral ischemia occurs, inflammatory cells will be activated, resulting in increased secretion. When the secretion of IL-1*β* increases, it will promote the activation of TNF-*α*, and both will promote the activation of other inflammatory cells, thereby aggravating brain damage [21]. IL-10 can reduce the expression of inflammatory cytokines, reduce the damage to neurons caused by neurotoxic substances produced by excitatory amino acids, and has a certain neuroprotective effect. This study found that high-dose volatile oil can decrease the contents of TNF-*α* and IL-1*β* (*P* < 0.01) and increase the contents of IL-10 in the serum (*P* < 0.01).

Oxidative stress is the main pathogenesis of secondary injury after cerebral ischemia reperfusion, and it is also one of the research hotspots in this field. Studies have shown that after cerebral ischemia, due to different cell types and production conditions of NO, NO plays the dual role of protection and injury. In the early stage of cerebral ischemia, the produced NO has the protective effect of dilating blood vessels and increasing blood flow in the brain, but it is only maintained within the first few hours, and it is mainly neurotoxic in the later stage. Therefore, inhibition of NO after cerebral ischemia can improve brain tissue damage and neuronal apoptosis [22]. SOD is an important peroxide-degrading enzyme. It is involved in the body's ischemic diseases, tumors, inflammation, and aging. SOD can effectively remove oxygen free radicals in the brain, thereby inhibiting the peroxidation of brain tissue and protecting brain tissue. The level of SOD activity can indirectly reflect the body's ability to scavenge excess oxygen free radicals [22]. Nerve cell damage will produce a large amount of lipid peroxide (MDA), so the MDA level reflects the degree of nerve cell damage. This study showed that medium- and high-dose of volatile oil can significantly increase the level of SOD and reduce the levels of MDA and NO in rat brain tissues (*P* < 0.01).

Bcl-2 and Bax proteins are a pair of endogenous proteins with the function of regulating cell apoptosis. Bax, as a transcription factor, is involved in the gene transcription process of inducing cell apoptosis, while Bcl-2 inhibits the function of Bax, which determines the cell survival [23]. Caspase-3 is a key regulatory molecule of the caspase cascade reaction. Cleaved caspase-3 is activated when stimulated by upstream apoptotic signals and then cleaved various cell substrates to perform apoptosis [24]. Our work suggests that high dose of volatile oil can decrease the level of cleaved caspase-3 and Bax protein and increase the levels of Bcl-2.

In summary, in this study, the therapeutic effect of VOFGA on the treatment of ischemic stroke with the combination of phlegm and blood stasis was studied. The results showed that the effects of VOFGA on lipid metabolism, anti-inflammation, antioxidation, and antiapoptosis were all found positive. It is suggested that the mechanisms of traditional Chinese medicine in the treatment of diseases are a multicomponent, multipathway, multitarget mode of action, which provides more ideas for the mechanism of VOFGA in the treatment of phlegm-stasis ischemic stroke and provides a broader research direction for future in-depth research.

## 5. Conclusions

In this study, a gas mass spectrometer was used to analyze and determine volatile oils, and 46 components were obtained. The high-dose VOFGA group demonstrated a reduced neurological damage and brain water content; improved whole blood viscosity and red blood cell aggregation index at various shear rates; reduced the levels of TG, TC, LDL-C, TNF-*α*, IL-1*β*, MDA, NO, Bax, and cleaved caspase-3; and increased the contents of HDL-C, IL-10, SOD, and Bcl-2. The findings suggest that the mechanism of FGA in treating cerebral ischemia caused by intermingled phlegm and blood stasis and improving blood rheology are related to lipid metabolism, anti-inflammatory, antioxidation, and antiapoptosis. In addition, the mucosal toxicity of VOFGA is relatively small, indicating that nasal administration is a safe and reliable method for VOFGA.

## Figures and Tables

**Table 1:** Main components in VOFGA.

Retention time	Name of component	Molecule formula	Relative molecular mass	Percentage content (%)
2.554	Hexane	C_6_H_14_	86.18	21.72
2.824	Methylcyclopentane	C_6_H_12_	84.16	1.58
3.206	Cyclohexane	C_6_H_12_	84.16	1.46
6.227	Hexanal	C_6_H_12_O	100.16	1.57
8.132	2-Hexenal	C_6_H_10_O	98.14	0.68
10.152	Heptanal	C_7_H_14_O	114.19	0.51
12.653	2-Heptenal, (Z)-	C_7_H_12_O	112.17	0.60
12.75	Benzaldehyde	C_7_H_6_O	106.12	1.39
14.913	Octanal	C_8_H_16_O	128.21	0.81
17.59	2-Nonenal, (E)-	C_9_H_16_O	140.22	0.83
19.689	1,6-Octadien-3-ol, 3,7-dimethyl-	C_10_H_18_O	154.25	3.31
19.899	Nonanal	C_9_H_18_O	142.24	3.32
21.683	(1r,4r)-(+)-camphor	C_10_H_16_O	152.23	1.28
22.527	2-Nonenal, (Z)-	C_9_H_16_O	140.22	0.74
23.306	3-Cyclohexen-1-ol, 4-methyl-1-(1-methylethyl)-	C_10_H_18_O	154.25	0.60
23.981	3-Cyclohexene-1-methanol, alpha, alpha 4-trimethyl-	C_10_H_18_O	154.25	2.42
24.727	Decanal	C_10_H_20_O	156.27	0.50
26.335	2,6-Octadienal, 3,7-dimethyl-, (Z)-	C_10_H_16_O	152.23	0.55
27.002	2,6-Octadien-1-ol, 3,7-dimethyl-, (Z)-	C_10_H_18_O	154.2493	0.60
27.295	(E)-2-Decenal	C_10_H_18_O	154.25	1.16
27.707	3,7-dimethyl-2,6-Octadienal	C_10_H_16_O	152.23	2.65
28.067	1,3,6-Heptatriene	C_7_H_10_	94.1543	1.04
29.769	(E,E)-2,4-Decadienal	C_10_H_16_O	152.23	1.71
30.181	2,4-dihydroxy-6-methyl-benzaldehyde	C_8_H_8_O_3_	152.15	4.07
30.89	2-Pentylcyclopentanone	C_10_H_18_O	154.2493	1.30
31.531	Eugenol	C_10_H_12_O_2_	164.2	0.74
31.756	Gamma-nonanolactone	C_9_H_16_O_2_	156.22	1.65
31.988	Benzene 4-ethenyl-1,2-dimethoxy-	C_10_H_12_O_2_	164.2	0.45
33.604	Methyleugenol	C_11_H_14_O_2_	178.23	1.67
34.526	Alpha-ionone	C_13_H_20_O	192.3	1.43
35.066	Paeonol	C_9_H_10_O_3_	166.18	5.20
35.568	Geranylacetone	C_13_H_22_O	194.31	0.55
36.921	Irisone	C_13_H_20_O	192.3	3.56
37.307	Octacosane	C_28_H_58_	394.76	0.57
37.806	3-fluoro-4-methoxy-acetophenone	C_9_H_9_FO_2_	168.16	2.04
38.087	2,4-Di-tert-butylphenol	C_14_H_22_O	206.32	2.29
38.346	1-Cyclopentyl-3-ethoxy-2-propanone	C_10_H_18_O_2_	170.2487	8.46
39.759	Elemicin	C_12_H_16_O_3_	208.25	1.57
40.423	Benzene, 1,2,4-trimethoxy-5-(1-propenyl)-, (Z)-	C_12_H_16_O_3_	208.25	0.46
41.371	Cedrol	C_15_H_26_O	222.37	0.66
49.434	2-Pentadecanone, 6,10,14-trimethyl-	C_18_H_36_O	268.4778	2.22
50.18	1-Ethynyl-1-cyclohexanol	C_8_H_12_O	124.18	0.94
51.009	Methyl hexadecanoate	C_17_H_34_O_2_	270.45	0.65
51.71	Palmitic acid	C_16_H_32_O_2_	256.42	4.69
53.846	9-(Z)-methyl octadecenoate	C_19_H_36_O_2_	296.4879	0.74
54.517	Oleic acid	C_18_H_34_O_2_	282.46	0.63

**Table 2:** Effects of VOFGA on neuroethology and cerebral edema (*n* = 5).

Groups	Dose (mg/kg)	Behavioral score	Brain water content (%)
Normal	—	0	70.61 ± 1.12
Sham	—	0	70.01 ± 1.27
Model	—	3.00 ± 0.00^*∗∗*^	86.09 ± 0.76^*∗∗*^
Nimodipine	5.0	0.20 ± 0.44^##^	72.74 ± 1.46^##^
VOFGA	0.0	3.00 ± 0.00	85.30 ± 1.89
VOFGA	0.077	2.00 ± 0.70^##^	79.59 ± 1.03^##^
VOFGA	0.154	1.00 ± 0.70^##^	75.23 ± 1.03^##^
VOFGA	0.308	0.40 ± 0.54^##^	72.43 ± 1.28^##^

Compared with the normal group,  ^*∗*^*P* < 0.05,  ^*∗∗*^*P* < 0.01. Compared with the model group, ^#^*P* < 0.05, ^##^*P* < 0.01.

**Table 3:** Effect of VOFGA on hemorheology in rats.

Groups	WBV (1/s) [RT1]	WBV (5/s) [RT5]	WBV (50/s) [RT50]	WBV (100/s) [CQ]	WBV (200/s) [RT200]	HJJ	KS
Normal	22.36 ± 6.64	9.68 ± 3.01	4.92 ± 1.64	4.4 ± 1.49	4.05 ± 1.39	5.77 ± 1.10	3.55 ± 0.30
Sham	80.23 ± 3.25^*∗∗*^	27.52 ± 1.18^*∗∗*^	10.05 ± 0.52^*∗∗*^	7.93 ± 0.41^*∗∗*^	7.27 ± 0.54^*∗∗*^	10.98 ± 0.62^*∗∗*^	6.96 ± 0.57^*∗∗*^
Model	79.04 ± 6.35^*∗∗*^	27.07 ± 1.71^*∗∗*^	10.03 ± 0.58^*∗∗*^	8.35 ± 0.51^*∗∗*^	7.26 ± 0.48^*∗∗*^	10.7 ± 0.86^*∗∗*^	6.98 ± 0.19^*∗∗*^
Nimodipine	27.42 ± 6.6^##^	11.86 ± 2.23^##^	6.03 ± 0.77^##^	5.39 ± 0.62^##^	4.96 ± 0.53^##^	5.47 ± 0.77^##^	3.99 ± 0.34^##^
VOFGA (0 mg/kg)	81.43 ± 31.5^*∗∗*^	26.52 ± 7.25^*∗∗*^	9.34 ± 1.34^*∗∗*^	7.9 ± 1.13^*∗∗*^	6.95 ± 0.99^*∗∗*^	10.7 ± 0.86^*∗∗*^	6.84 ± 0.31^*∗∗*^
VOFGA (0.077 mg/kg)	22.83 ± 1.4^##^	10.66 ± 0.56^##^	6.83 ± 0.71^##^	6.11 ± 0.27^##^	5.49 ± 0.16^##^	8.22 ± 0.79^##^	4.24 ± 0.58^##^
VOFGA (0.154 mg/kg)	31.21 ± 2.92^##^	13.07 ± 0.96^##^	6.37 ± 0.34^##^	5.65 ± 0.28^##^	5.17 ± 0.25^##^	6.03 ± 0.35^##^	4.08 ± 0.18^##^
VOFGA (0.308 mg/kg)	31.18 ± 10.98^##^	12.91 ± 3.34^##^	6.42 ± 0.92^##^	5.52 ± 0.69^##^	5.05 ± 0.55^##^	6.06 ± 1.50^##^	3.98 ± 0.27^##^

Compared with the normal group,  ^*∗*^*P* < 0.05,  ^*∗∗*^*P* < 0.01. Compared with the model group, ^#^*P* < 0.05, ^##^*P* < 0.01.

## Data Availability

The data that support the findings of this study are available on request from the first author Chengyue Luo.
